# Scanning electron microscopy assessment of the Descemet membrane interface during DMEK graft preparation

**DOI:** 10.1038/s41598-017-18991-7

**Published:** 2018-01-11

**Authors:** Javier Cabrerizo, Thomas Forshaw, Clara Rodriguez-Aierbe, Jesus Garrido-Fierro

**Affiliations:** 1 Department of Ophthalmology, Rigshospitalet/Glostrup, University of Copenhagen, Copenhagen, Denmark; 2 Copenhagen Eye Foundation, Copenhagen, Denmark; 3 Department of Ophthalmology, Zealand University Hospital, Roskilde, Denmark; 4 Faculty of Health and Medical Sciences, University of Copenhagen, Copenhagen, Denmark; 5 Basque Centre for Transfusions and Human Tissue, Galdakao, Spain; 6 Department of Ophthalmology, University Hospital of Alava, Vitoria -Gasteiz, Spain

## Abstract

We set out to determine microscopic characteristics of the Descemet membrane interface during Descemet membrane endothelial keratoplasty (DMEK) graft preparation. Ten corneas were partially prepared, preserving half of the Descemet membrane attached to the stroma to enable visualisation of the Descemet-stroma interface. This tissue was prepared for viewing with a scanning electron microscope. The Descemet-stroma interface was categorised into three regions: centre, mid-periphery and periphery. We classified adhesions in these regions as either minor thread-like adhesions or major bridge-like adhesions with stromal detachments. We found a region-specific differentiation of the Descemet-stroma morphology. The presence of minor *(P* = *0*,*0001)* and major *(P* = *0*,*0001)* adhesions at the explored regions of the Descemet-stroma interface were found to be statistically significant. Fibrotic linear adhesions were predominant in the centre and mid-periphery, whereas the larger bridge-like adhesions were found mainly in the periphery. In addition, we observed a positive correlation between the size of the adhesions and the presence of ruptures in the underlying stromal bed. Viewing of the Descemet-stroma interface with electron microscopy reveals morphological differences between the centre of a graft and its periphery. These findings are of potential clinical relevance in terms of developing a better understanding of tissue behaviour during graft preparation.

## Introduction

Descemet membrane endothelial keratoplasty (DMEK), first reported by Melles in 2009^1^, is notable for enabling a complete anatomical restoration of the corneal architecture by only transplanting donor corneal endothelium and Descemet membrane (DM). Technical difficulties aside, DMEK seems to bring advantages in terms of visual rehabilitation^2^ and rejection rates^3^ compared with previous techniques^4^. DMEK may also represent an opportunity for a more efficient tissue management as only the DM and endothelium are used, whereas the remaining anterior cornea is left available for anterior lamellar keratoplasty^5^, allowing two grafts to be taken from a single donor eye.

Reported rates of unsuccessful or suboptimal DMEK graft preparations are low, but nevertheless persist. A centralised eye bank based preparation is currently preferred by most large centres to minimize the loss of tissue due to failed graft preparation^6^. Deeper understanding of both the histological and logistical aspects involved in DMEK preparation may enable outcomes that are more predictable and a subsequent decrease in tissue loss.

Eye banks use several donor characteristics that influence DMEK graft preparation when selecting donors. Donor age is linked to DM thickness and preparation success rate^7^, while diabetes mellitus and glaucoma are associated with adverse outcomes^8^. In the setting of the operating theatre, mechanical damage to the donor graft is regularly encountered as a result of tissue ‘breaks’ or tissue ‘tears’ that influence the scrolling and unfolding of the graft as well as subsequent surgical outcomes. Although donor demographics and comorbidities are known to determine tissue structure and graft preparation, the corneal histology responsible for unsuccessful DMEK graft preparation remains poorly understood. In this report, we examine the ultra-structural characteristics of the Descemet–stroma interface (DSI) during DMEK graft preparation as a means to explain tissue behaviour during graft preparation.

## Material and Methods

### Ethical statement

This study was approved by the regulatory authority, Comite Ético de Investigación Clínica (CEIC) and adheres to the tenets of the Declaration of Helsinki. The authors confirm that all methods were carried out in accordance with the guidelines and regulations of the competent authority, Comite Ético de Investigación Clínica (CEIC). The subjects involved in the study provided their informed consent.

### Sampling

**Figure 1:**
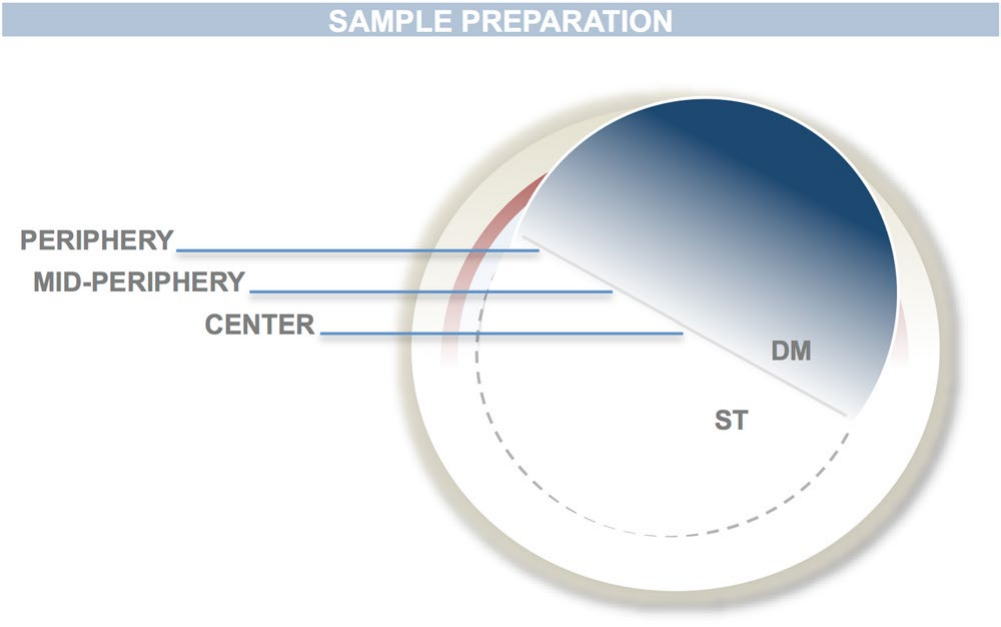
Schematic illustration of the sample preparation showing the partially detached DM exposing 11 mm of DSI along the center of the cornea. The DSI was divided in 3 distinctive regions for qualitative and quantitative SEM analyses. Center: 0–2 mm from the centre of the cornea. Mid-periphery: 2–4 mm from the center of the cornea. Periphery: 4–5.5 mm form the centre of the cornea.

We used five human corneas from the The Basque Transfusion and Human Tissue Centre and five human corneas from the Danish Cornea Bank. Corneoscleral rims were excised and stored in corneal culture medium (Tissue-C, Alchimia, Ponte S. Nicolò, Italy) at 31 °C within one week post-mortem. Endothelial cell density and morphology were examined by specular microscopy (CellChek D, Konan Medical, Irvine, USA). Each cornea had an intact DM and normal morphology prior to the preparation. Corneas were marked as unsuitable for transplantation due to lower central endothelial cell density, under 2200 cells/mm^2^. DMEK preparation was performed as previously described^9^ until half of the DM was detached (Supplementary material: Video 1.) The detached DM was folded backwards allowing the DSI to be exposed (Fig. 1).

### Scanning electron microscopy

Due to the size and structural characteristics of the samples, we used scanning electron microscopy for DSI visualisation. Samples were fixed with 2% glutaraldehyde in 80 mM sodium cacodylate buffer (pH 7.2–7.4, 320–340 mOsm/kg), processed with gold-coated magnetic particles for solid-phase immunoassays and, after critical point of drying, examined with a scanning electron microscope (SEM) (JEOL JSM-7000F, JEOL, Peabody, USA: first five corneas. FEI Quanta 3D: last five corneas). The DSI interface was divided into three regions: centre (0–2 mm from the centre), mid-periphery (2–4 mm from the centre) and periphery (4–5.5 mm from the centre) (Fig. 1). Each region was systematically studied in every sample. Firstly, the samples were screened at low magnification x30–x200 to identify morphological changes or adhesions in the DSI. Once identified, pictures at higher magnifications (x300–x60.000) were taken of these regions to further study and classify the morphology of the adhesions.

The morphological findings were classified as either minor thread-like adhesions or major bridge-like adhesions. Minor thread-like adhesions are fibrillary-elongated structures attached to the DM that originate from the surface of the underlying stroma. Major bridge-like adhesions are larger structures, with a wide attachment to the DM related to a large stromal pre-descemetic detachment or rupture.

### Statistical analyses

SPSS 22 was used for statistical analyses of the data. *Chi-Square* Test and nominal regression were used to assess correlation between corneal region and type of adhesion.

## Results

### Demographics

All corneas were stored in organ culture for an average of 70,7 days before preparation and tissue fixation. Donor age average was 74,8 years, converging with previously reported donor series^10^ (Table 1). 4 donor subjects had been diagnosed with hypertension (HTN) and were without any of the other reported conditions. 1 donor subjects had been diagnosed with hypercholesterolaemia (HC) and/or hyperlipidaemia (HLP) and were without any of the other reported conditions. 1 donor subjects had been diagnosed with HTN, HLP/HC and diabetes mellitus type 2 (T2DM).

**Table 1:** Demographics and SEM morphological classifications of the 10 donor corneas. The presence of one or more adhesions in every region of the DSI was reported.

CASE	DAYS TO PREPARATION	AGE	GENDER	CENTER		MID-PERIPEHRY		PERIPHERY	
				MINOR ADHESIONS	MAJOR ADHESIONS	MINOR ADHESIONS	MAJOR ADHESIONS	MINOR ADHESIONS	MAJOR ADHESIONS
Cornea 1	29	74	MALE	NO	NO	YES	NO	NO	YES
Cornea 2	5	82	FEMALE	NO	NO	YES	NO	NO	NO
Cornea 3	9	73	FEMALE	NO	NO	YES	NO	NO	YES
Cornea 4	8	59	MALE	NO	NO	YES	NO	NO	YES
Cornea 5	26	86	MALE	NO	NO	YES	NO	NO	YES
Cornea 6	132	74	MALE	NO	NO	YES	NO	NO	YES
Cornea 7	132	74	MALE	NO	NO	YES	NO	NO	YES
Cornea 8	130	80	FEMALE	NO	NO	YES	NO	NO	NO
Cornea 9	120	84	MALE	NO	NO	YES	NO	NO	YES
Cornea 10	116	62	MALE	NO	NO	YES	NO	NO	YES

### Scanning electron microscopy

**Figure 2:**
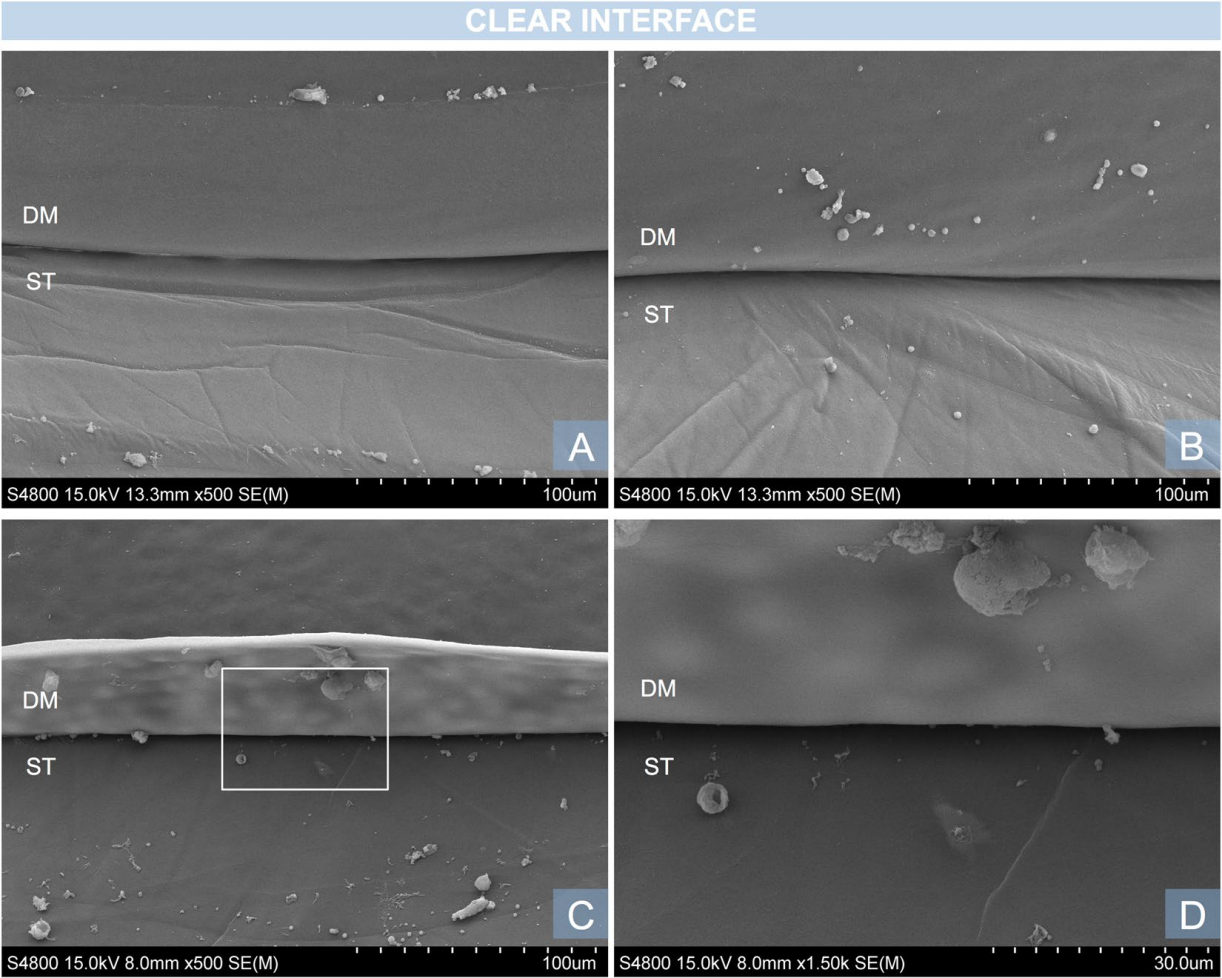
Examples of clear DSI, characteristic of the centre of the DSI. (**A**,**B**) Magnification x500. Descemet membrane (DM) and underlying stroma (ST) with folds. Both surfaces are regular and smooth and the DSI appears as a distinctive continuous dark line. (**C**,**D**) A similar image of another sample with a less oedematous underlying stroma. The white square in C is represented in larger magnification (x1.500) in D. Note the circular printings of the endothelial cell nuclei in the DM.

**Figure 3:**
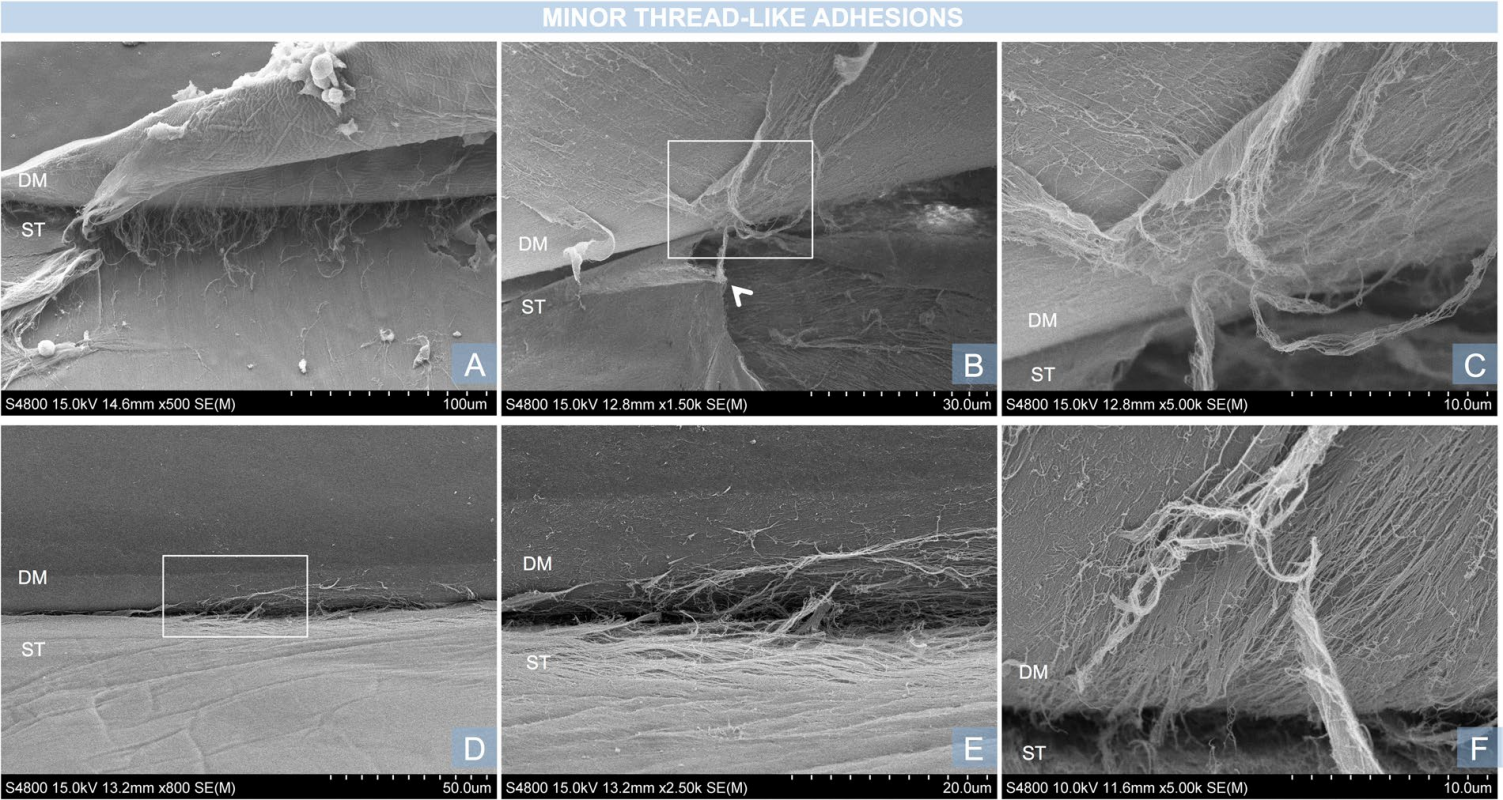
Examples of long, thread-like adhesions, characteristic of the mid-periphery of the DSI. (**A**) Magnification x500. Group of thin adhesions without stromal disruption. (**B**) Magnification x1.500. Thread-like adhesion with underlying stromal rupture. Note the attachment to the edge of the stromal rupture (white arrow). The white square in B is represented in larger magnification in C. (**C**) Large magnification (x5.000) of the thread-like adhesion shows the fibres attached to the DM. (**D**) Magnification x800. Large area of thread-like adhesions with ruptures of the underlying stroma. The white square in D is represented in larger magnification in E. (**E**) Magnification x2.500. Note how the direction of the stromal ruptures correlate with the orientation of the collagen fibres in the deep stroma. (**F**) Magnification x5.000. Stromal fibres involved in a thread-like adhesion.

**Figure 4:**
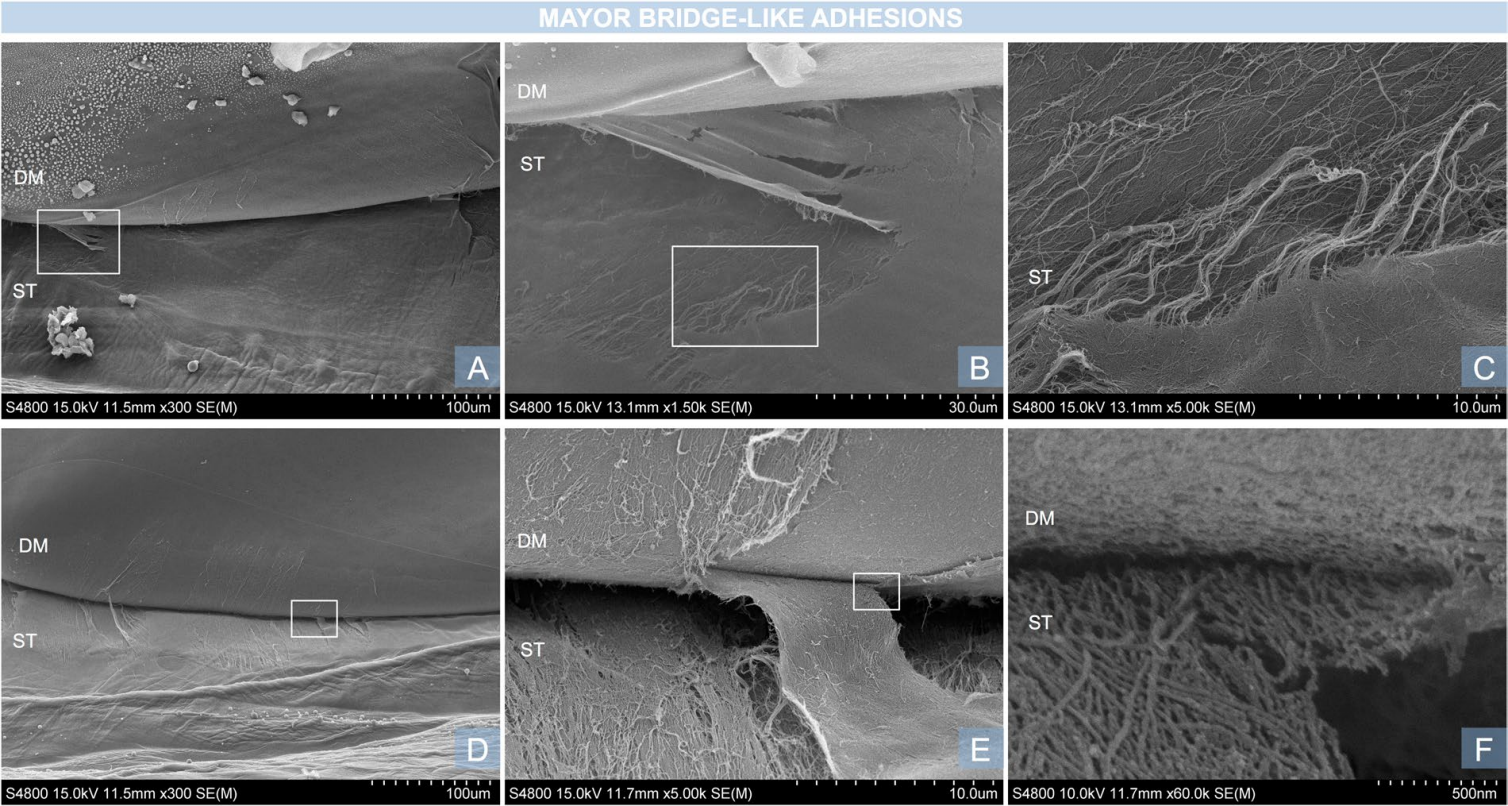
Examples of broad, bridge-like adhesions, characteristic of the periphery of the DSI. (**A**) Magnification x300. Bridge-like adhesions with large ruptures in the underlying stroma. The white square in A is represented in larger magnification in B. (**B**) Large magnification (x1.500) of a bridge-like adhesion. The underlying stroma shows a detachment of the deep layer. The white square in B is represented in larger magnification in C. (**C**) Large magnification (x5.000) of a stromal rupture. Fibres under the rupture show a different, less structured configuration. The borders appeared irregular. (**D**) Magnification x300. Another example of the periphery of the DSI showing multiple bridge-like adhesions. The white square in D is represented in larger magnification in E. (**E**) Large magnification (x5.000) of a bridge-like adhesion with a broad attachment to the DM. The white square in E is represented in larger magnification in F. (**F**) Large magnification (60.000) of the interface between the adhesion and the DM.

**Figure 5:**
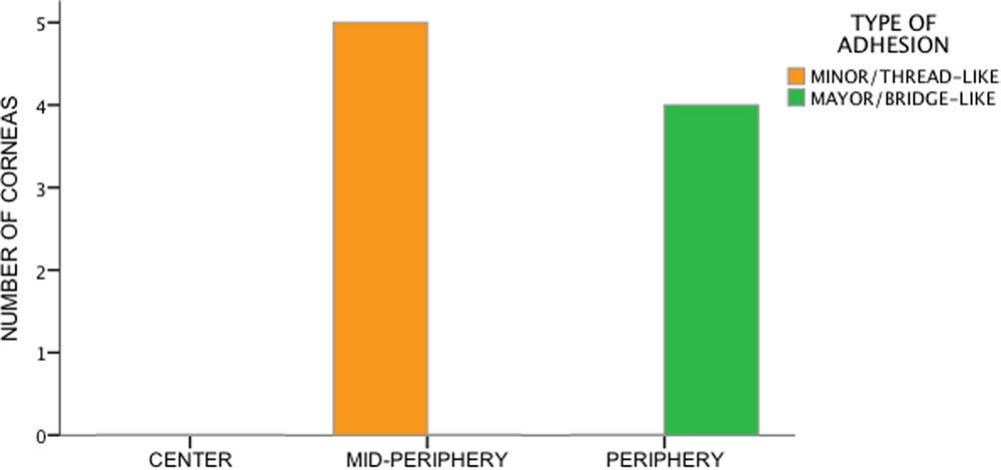
Graph showing the prevalence of the two different types of adhesions in the different regions of the DSI. None of the corneas demonstrated adhesions in the central DSI. All ten corneas demonstrated at least one minor/thread-like adhesion in the mid-periphery (orange bar). No major/bridge-like adhesions were found in the mid-periphery. Eight corneas presented at least one major/bridge-like adhesion in the periphery (green bar). No minor/thread-like adhesions were found in the periphery.

The DSI typically appears as a straight, black line between the DM and the stroma when viewed with an SEM. The DSI was successfully visualised in all samples. There were differences in the morphology and ultra-structural characteristics of the DSI in all three regions studied. The centre of the DSI did not show adhesions, DM ruptures or stromal irregularities in any of the ten samples. The DM and underlying stroma appeared intact, with a smooth surface as shown in Fig. 2. All corneas showed minor thread-like adhesions (Fig. 3) in the mid-periphery. These were clustered together and were limited to the area of the mid-periphery. At high magnification they appeared as a small group of fibres attached to the DM. No major bridge-like adhesions were found in the mid-periphery (2–4 mm from the centre). Major bridge-like adhesions were present in the periphery of eight corneas (Fig. 4). Major bridge-like adhesions were isolated and related to a large rupture of the underlying stroma. Authors observed a trend towards the extreme periphery (5–5.5 mm), where major bridge-like adhesions were larger and more common (Fig. 5).

### Statistical analyses

Chi-square test shows dependence between the presence of minor *(P* = *0*,*0001)* and major *(P* = *0*,*0001)* adhesions and the explored regions of the DSI^11^. Nominal regression analyses show location as an outstanding predictor for the presence and the type of adhesions, with the statistically significant model fitting *(P* < *0*.*000)*.

## Discussion

This report details structural findings in the DSI on scanning electron microscopy during graft preparation. These findings consist of two different morphologies that are specific to region and may have potential implications for graft tearing during preparation.

The mechanical properties of the DM have grown increasingly important as endothelial keratoplasty has evolved into DMEK, requiring the manual preparation of very thin grafts. Uniaxial stress (σ) can be expressed as σ = Force/Area^12^. This concept acquires special relevance during graft preparation in DMEK, where the 10 to 12 μm thick DM is subject to stretch and traction forces that can potentially spoil the graft. These forces distribute evenly in the case of a regular interface, but concentrate in areas that provide greater resistance, with tissue adhesions creating stress lines along the tissue^13^ and affecting cellular junctions^14^. Tissue structural stress increases when adhesions are greater in size and number, causing DM to tear when the membrane’s tensile strength has been overcome. The identification of the DM elastic modulus^15^ and the description of changes in the mechanical properties of the DM related to Fuchs Corneal Endothelial Dystrophy^16^ have given insight into the resistance of the DM to traction stress during DMEK graft preparation. However, this alone is not enough to explain the variability in DM characteristics by donor and by region.

Connections between the DM and the posterior stroma have been previously identified by transmission electron microscopy and inmunohistochemistry^17^. These have been described as posterior stromal fibres projecting to the extracellular matrix of the DM, the prevalence and size of which may vary between individuals^10^ and may contribute to optical differences between the central and the peripheral cornea. Our study dovetails with these findings showing the morphology of DSI adhesions during a dynamic surgical moment, displaying their role in DMEK graft preparation. These adhesions create high traction points in the DM, proving that the advance of the cleavage plane does not occur evenly in every region of the DSI. During preparation, adhesions may resolve spontaneously after a stromal rupture, or create a DM rupture when their adhesion tensile strength is greater than that of the DM, causing tearing of the graft. From our image analyses, we hypothesised that this is more likely to happen in large bridge-like adhesions, which are morphologically wider and predominate in the periphery of the DSI.

With increasing numbers of small and mid-sized centres willing to shift towards DMEK, meeting the need for viable DMEK grafts has become a major concern for eye banks that are transitioning from being tissue storing facilities to tissue laboratories. The key to providing grafts of a consistently high standard with minimal tissue loss may lie with a better understanding of the factors that lead to DM tearing; for example: donor medical history, tissue harvesting and choice of preparation technique. Understanding the DSI morphological features that trigger tearing of the DM creates opportunities to refine preparation techniques.

Firstly, our morphological classification allows for a systematic approach to quantification and grading of the DSI adhesions. This enables individuals to objectively describe preparation difficulty independently of a surgeon’s opinion or operator-specific outcomes. This opens up new possibilities in DSI grading in donors with different medical conditions and in samples that undergo different storage methods.

Secondly, the distinct regional differences described in our series can help decision making during DMEK preparation, such as whether to score or avoid the periphery of the cornea in difficult cases. Furthermore, future research into the development of new, DMEK preparation-specific instruments may allow visualisation of adhesions and tissue traction during DMEK preparation. Our findings may also explain the usefulness of removing the trabecular meshwork and Swalbe’s Line prior to pulling the membrane, avoiding the peripheral DSI and the potentially higher risk of tearing associated with it^7^.

Corneas were stored in culture medium for less than one month, and at the time of preparation the samples showed no evidence of stromal scar or thinning, or any areas of absent endothelial cover in the Descemet membrane. Despite the strengths of this study, namely: distinct morphological features, standardised handling and storage conditions and representative donor demographics; a limitation of this study is that it includes only 10 different samples. Variations stemming from different donor demographics should be expected, offering a future field of research.

In conclusion, our work provides a classification of DSI adhesions by region during graft preparation and gives insight into the dynamic interactions of stroma and DM. Moreover, our findings may play an important role in successful graft preparation and also provide opportunities for further research into the structural properties of corneal tissue.

## Electronic supplementary material

Video 1
